# Vaspin Increases Nitric Oxide Bioavailability through the Reduction of Asymmetric Dimethylarginine in Vascular Endothelial Cells

**DOI:** 10.1371/journal.pone.0052346

**Published:** 2012-12-28

**Authors:** Chang Hee Jung, Woo Je Lee, Jenie Yoonoo Hwang, Min Jung Lee, So Mi Seol, Yun Mi Kim, Yoo La Lee, Hyun Sik Kim, Min-Seon Kim, Joong-Yeol Park

**Affiliations:** 1 Department of Internal Medicine, University of Ulsan College of Medicine, Seoul, Republic of Korea; 2 Asan Institute of Life Sciences, University of Ulsan College of Medicine, Seoul, Republic of Korea; University Hospital Freiburg, Germany

## Abstract

Vaspin is an adipocytokine recently identified in the visceral adipose tissue of diabetic rats and having anti-diabetic effects. We have recently shown that vaspin has anti-atherogenic effect through Akt-mediated inhibition of endothelial cell apoptosis. Decreased activity of endothelial nitric oxide synthase (eNOS) plays an important role in the pathogenesis of atherosclerosis. Asymmetric dimethylarginine (ADMA) is a well-known endogenous competitive inhibitor of eNOS and risk factor of cardiovascular diseases. The aim of this study was to examine whether vaspin might protect against atherosclerosis through its beneficial effects on the ADMA-eNOS system. Treatment of vaspin significantly increased NO secretion from endothelial cells and isolated aorta from Sprague-Dawley (SD) rats. Furthermore, treatment of vaspin prevented fatty acid-induced decrease in endothelium-dependent vasorelaxation in isolated aorta of SD rat. For the mechanism of vaspin-induced NO biosynthesis, vaspin activated the STAT3 signaling pathway and stimulated eNOS phosphorylation (Ser 1177), a marker of eNOS activation, through STAT3-dependent mechanism. Furthermore, vaspin treatment increased the expression of dimethylarginine dimethylaminohydrolase (DDAH) II, the responsible enzyme for the degradation of ADMA, leading to a reduction in ADMA levels. Vaspin-induced increase in DDAH II gene expression was through STAT3-mediated stimulation of DDAH II promoter activity. These results suggest that vaspin increases eNOS activity by reducing ADMA level through STAT3-mediated regulation of DDAH II expression. Our findings provide a novel molecular mechanism of antiatherogenic actions of vaspin.

## Introduction

Nitric oxide (NO) synthesized by endothelial nitric oxide synthase (eNOS) plays a crucial role in the maintenance of vascular tone and structure [1]. Decreased NO bioavailability is one of the major features of cardiovascular diseases (CVD) [2]. An impairment of endothelium-dependent vasorelaxation is present in atherosclerotic vessels even before vascular structural changes occur and represents the reduced eNOS-derived NO bioavailability [3]. Endothelial dysfunction characterized by an impairment of endothelium dependent vasorelaxation, and thereby reduced eNOS-derived NO bioactivity, is the critical step for atherogenesis [3].

Asymmetric dimethylarginine (ADMA), which derives from methylation of arginine residues in proteins and is metabolized to citrulline and dimethylamine by the enzyme dimethylarginine dimethylaminohydrolase (DDAH), is an endogenous competitive inhibitor of eNOS [4]. ADMA has been shown to be a cardiovascular risk factor for numerous diseases [5] and elevated ADMA levels have been shown to attenuate endothelium-dependent vasodilation in humans [6]. In addition, dysregulation of DDAH has been shown to be a mechanism for endothelial dysfunction in hypercholesterolemia or diabetes [7], [8]. Collectively, the ADMA-DDAH system can be a target for treating CVD through the regulation of endogenous NO bioavailability.

Adipose tissue is now regarded as not only a tissue for the deposit of extra energy, but also as an active endocrine organ which can release a variety of cytokines termed adipocytokines. Vaspin (visceral adipose tissue-derived serine protease inhibitor), a member of the serine protease inhibitor family, is a novel 392–395 amino acid adipocytokine identified in visceral white adipose tissues of the Otsuka Long-Evans Tokushima fatty (OLEFT) rat, an animal model of abdominal obesity with type 2 diabetes [9]. Although it was reported that vaspin might exert anti-inflammatory effects because it inhibits expression of proinflammatory adipocytokines including resistin, leptin and TNF-α in murine WAT [9], the exact function of vaspin in the body is largely unknown, especially in vascular cells. Recently, we have found that it has antiatherogenic properties by preventing free fatty acid (FFA)-induced endothelial apoptosis through its stimulatory effect on the phosphatidiylinositol 3-kinase/Akt pathway, a representative insulin signaling pathway [10].

The aim of study was to examine whether vaspin might protect against atherosclerosis through its beneficial effects on the ADMA-eNOS system in vascular endothelial cells and,if so, to investigate the molecular mechanism underlying these effects.

## Materials and Methods

### Cell Culture, Treatment and Transfection of siRNA

HAECs were obtained from Lonza Inc. (Walkersville, MD, USA) and maintained in endothelial basal medium (EBM-2; Lonza) supplemented with various growth factors required for the growth of endothelial cells and 2% fetal bovine serum (FBS) at 37°C in a humidified incubator supplemented with 5% CO_2_. In all experiments, cells were used at six or fewer passages.

Vaspin was obtained from Adipogen Inc. (Incheon, Korea), and DMSO was used as vehicle. Cells were transferred to medium containing 1% FBS and incubated in media containing a various concentrations of vaspin for the indicated time. We used all-trans-retinoic acid (atRA, Sigma, St.Louis, MO, USA), a DDAH activator [11], as a positive control.

We used a small interfering RNA specific to human signal transducer and activator of transcription 3 (STAT3 siRNA, QIAGEN, Valencia, CA, USA), DDAH II (Bioneer, Daejeon, Korea) or non-targeting scrambled siRNA (control siRNA, QIAGEN for STAT3 and Bioneer for DDAH II) to examine whether STAT3 or DDAH II mediated the vaspin-induced changes in the eNOS activity in endothelial cells as well as the levels of NO and ADMA in cultured media of endothelial cells. The sequences for STAT3 and DDAH II are 5′-CAG CCT CTC TGC AGA ATT CAA-3′, and 5′-CCG AAU UGU GGA AAU AGG A-3′, respectively. For the experiments using siRNA, 100 nM of STAT3 siRNA, DDAH II siRNA or control siRNA was transfected into HAECs using LipofectAMINE 2000 (Invitrogen, Carlsbad, CA, USA) 24 hr before vaspin treatment.

### Determination of ADMA and NO Concentration

Concentrations of ADMA and NO in the conditioned media were measured by enzyme-linked immunosorbent assay (ELISA) kit (Immunodiagnostik AG, Benshim, Germany for ADMA, and Enzo Life Science, NY, USA for NO, respectively) according to the kit supplier’s instructions. Nitro-L-arginine (L-NNA, Sigma), an eNOS inhibitor [12], was used as a negative control.

### Western Blotting Analysis

Protein expression in cells and tissues was measured by Western blot analysis as previously described [13]. The following primary antibodies were used: anti-phospho eNOS (Ser1177) (1∶1000, BD Bioscience, San Jose, CA, USA), anti-eNOS (1∶1000, Cell Signaling, Danvers, MA, USA), anti-phospho vasodilator-stimulated phosphoprotein (VASP, Ser239) (1∶1000, Bioworld Technology, St. Louis Park, MN, USA), anti-VASP (1∶1000, Bioworld Technology), anti-phospho STAT3 (Tyr705) (1∶1000, Cell Signaling), anti-STAT3 (1∶1000, Cell Signaling), and anti-β-actin (1∶10000, Sigma). After incubating with primary antibodies, membranes were washed and incubated with horseradish peroxidase-conjugated secondary antibodies (Vector laboratories Inc. Burlingame, CA, USA). Immunoreactive bands were visualized by enhanced chemiluminescence (Amersham Bioscience, UK).

### Real Time Polymerase Chain Reaction (PCR) Analysis

The mRNA expression of STAT3 and DDAH II was measured by real-time PCR analysis. (Methods S1).

### In vitro Transient Transfection and Reporter Gene Assays

DDAH II promoter-reporter plasmid was constructed by inserting DDAH II 5′ upstream fragment (from −1425 bp to 1 bp) into a pGL3-basic vector. Putative STAT3-binding site (from −1187 bp to −1179 bp) in DDAH II promoter was mutated by PCR overlap extension from 5′-TTT CGG CAA-3′ to 5′-CCC AGG CAA-3′. DDAH II promoter and its mutant were confirmed by sequencing. Cells were transfected with 200 ng of STAT3-expression vector (pUNO1-hSTAT3b, InvivoGen, San Diego, CA, USA), or 200 ng of wild type or mutant type reporter gene using LipofectAMINE 2000 reagent (Invitrogen). pRL-SV40 renilla luciferase control reporter vectors (10 ng, Promega, Madison, WI, USA) were co-transfected as an internal control. Twenty-four hours after the transfection, the cells were left unstimulated or stimulated with 100 ng/ml of vaspin and were harvested 16 h later. Luciferase activity was measured using a Dual-Luciferase Reporter Assay (Promega) and normalized to renilla luciferase activity.

### Electrophoretic Mobility Shift Assay (EMSA)

EMSA was used to determine whether vaspin increased the DNA-binding activity of STAT3.

We used a commercially available EMSA kit (Panomics, Inc., CA, USA). Briefly, this EMSA kit used streptavdin-HRP conjugate to detect a biotin-labeled transcription factor or unlabeled form (cold probe). The sequence for STAT3 probe (Panomics) was 5′-GAT CCT TCT GGG AAT TCC TAG ATC-3′ [14]. We used 2 ug or 4 ug of STAT3 Ab (SantaCruz Biotechnology, Inc., Santa Cruz, CA, USA) to detect supershift. The bands were visualized using enhanced chemiluminescence (Amersham Bioscience) after exposure to film.

### Animals

Eight-week-old male Sprague-Dawley (SD) rats (Orient, Sungnam, Korea) weighing 250 to 300 g were housed in cages containing 4 rats per cane and allowed ad libitum access to water and food. All experimental procedures were approved by the Institutional Animal Care and Use Committee of Asan Institute of Life Sciences.

### Ex vivo Measurement of Endothelium-dependent Vasorelaxation

Endothelium-dependent vasorelaxations were measured using an isometric force displacement transducer (Hugo Sachs Elektronik KG D-7806) as described previously [13]. The thoracic aorta was excised from SD rats after euthanization with pentobarbital, and then cleaned to remove fat and adhering tissue. The vessel was cut into several individual ring segments 2–3 mm in width, and suspended from a force-displacement transducer in a tissue bath. Ring segments were washed in Krebs-Henseleit buffer (118 mM NaCl, 4.6 mM KCl, 27.2 mM NaHCO_3,_ 1.2 mM KH_2_PO_4_, 1.2 mM MgSO_4_, 1.75 mM CaCl_2_, 0.03 mM Na_2_EDTA, and 11.1 mM glucose) maintained at 37°C and aerated with 95% O_2_ and 5% CO_2_. The tension was measured with an isotonic force diaplacement transducer (Hugo Sachs Elektronik KG D-7806) and recorded using a polygraph (Graphtec Linerecorder mark 8 WR3500). Vaspin (100 ng/ml) was pretreated for 4 hrs before the exposure to 100 µmol/L of linoleic acid (LA, Sigma) for 2 hr. The sub-maximal contraction of the aortic ring was induced by treatment with 300 µmol/L phenylephrine. When vascular tension reached a plateau, acetylcholine (from 10^−9^ to 10^−5^ mol/L) was added serially to the bath to induce endothelium-dependent vasorelaxation. Vascular relaxation data were calculated as the percentage of the maximal vasorelaxation, and the dose-response profile for each experiment was analyzed [15].

### Statistical Analysis

All data are shown as means ± standard error of mean (SEM). Comparisons among two groups or multiple groups were performed by independent student t-test or one-way ANOVA followed by a post hoc analysis using the Tukey’s multiple comparison test, respectively. The significance of differences in vascular relaxation between the 4 experimental groups was assessed by ANOVA with repeated measures. A *p-*value <0.05 was considered statistically significant. All experiments were performed at least three times. All statistical *analyses* were performed using *SPSS*11.5 for Windows (*SPSS*, Inc., Chicago, IL, USA).

## Results

### Effects of Vaspin on the Level NO and the Expression of eNOS and VASP in HAECs

**Figure 1:**
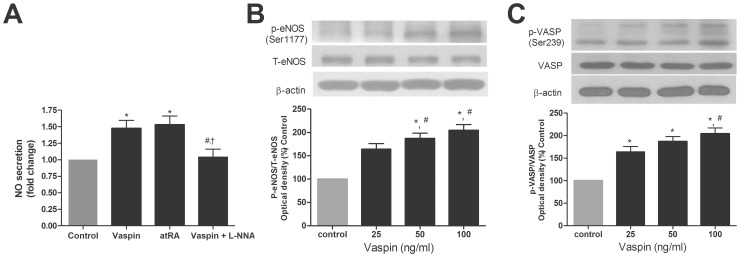
Vaspin increases the level of NO in conditioned media of HAECs and eNOS activity in HAECs. A. Effect of vaspin and atRA on NO levels in conditioned media of HAECs. HAECs were treated with vaspin (100 ng/ml) and atRA (1 µM) for 24 hr. atRA was used as a positive control. L-NNA (500 µM) was treated 30 min before vaspin treatment. **B.** Effect of vaspin on eNOS phosphorylation at serine 1177 residues in HAECs. HAECs were treated with indicated concentrations of vaspin for 16 hr. **C.** Effect of vaspin on VASP phosphorylation at serine 239 residues in HAECs. HAECs were treated with indicated concentrations of vaspin for 16 hr. Data are shown as means ± SEM of at least three independent experiments. In A: ^*^ *p*<0.05 vs. untreated (Control);^ #^ *p*<0.05 vs.vaspin group; ^†^ *p*<0.05 vs. atRA group. In B and C: ^*^ *p*<0.05 vs. untreated (Control); ^#^ *p*<0.05 vs. 25 ng/ml of vaspin group. NO, nitric oxide; atRA, all-trans-retinoic acid; L-NNA, Nitro-L-arginine; eNOS, endothelial nitric oxide synthase; VASP, vasodilator-stimulated phosphoprotein.

Compared to control cells, incubation of HAECs with 100 ng/ml of vaspin for 24 h increased the NO level by approximately 50% in conditioned media (Figure 1A). This effect of vaspin on NO was comparable to that of atRA (1 µM), a well-known DDAH activator [11] (Figure 1A). The pretreatment of L-NNA abolished this effect of vaspin on NO (Figure 1A).

To investigate whether vaspin had an effect on the level of NO through the regulation of eNOS activity, we first evaluated the effect of vaspin on the protein expression levels of total and phosphorylated (serine 1177)-eNOS in HAECs. Serine 1177 residue is known as a key phosphorylation site that positively regulates eNOS enzyme activity [16]. Incubation of HAECs with various concentration of vaspin ranged from 25 to 100 ng/ml for 16 h induced a steady increase in eNOS phosphorylation at serine 1177 residue (Figure 1B). Total eNOS expression was unaltered by vaspin over the treatment course of these studies.

Given that vaspin increased the activity of eNOS represented by serine site’s phosphorylation of eNOS, we next examined the change of VASP as a second messenger in response to the treatment of vaspin [17]. As shown in Figure 1C, incubation of HAECs with various concentration of vaspin ranged from 25 to 100 ng/ml for 16 h induced a steady increase in the phosphorylation of VASP (Figure 1C). Total VASP expression was unaltered by vaspin over the treatment course of these studies.

### Effects of Vaspin on the Level of NO and the Expression of eNOS in Vascular Tissue

**Figure 2:**
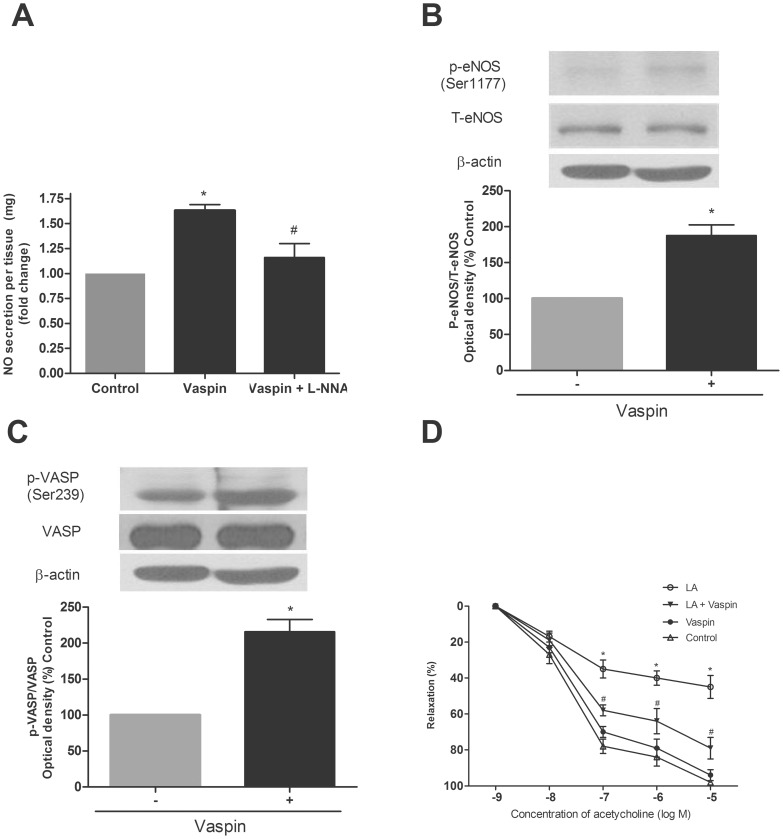
Vaspin increases the level of NO and eNOS activity in vascular tissues and restored the LA-induced impairment in vasorelaxation. A. Effect of vaspin on NO levels in conditioned media of vascular tissues. Vascular tissues were treated with vaspin (100 ng/ml) for 24 hr. L-NNA (500 µM) was treated 30 min before vaspin treatment. The fold changes were standardized according to the weight (mg) of vascular tissues of each group. **B–C.** Effects of vaspin on the phosphorylations of eNOS (B) and VASP (C) in vascular tissues. Vascular tissues were treated with vaspin (100 ng/ml) for 16 hr. **D.** Effect of vaspin (100 ng/ml) on LA-induced (100 µmol/L) impairment in vasorelaxation. Endothelium-dependent vasorelaxation was measured by the method described in Materials and Methods. Data are shown as means ± SEM of at least three independent experiments. In A: ^*^ *p*<0.05 vs. untreated (Control);^ #^ *p*<0.05 vs.vaspin group. In B and C: ^*^ *p*<0.05 vs. untreated (Control). In D: ^*^ *p*<0.05 vs. untreated (Control), ^#^ *p*<0.05 vs.LA alone group. NO, nitric oxide; L-NNA, Nitro-L-arginine; eNOS, endothelial nitric oxide synthase; VASP, vasodilator-stimulated phosphoprotein; LA, linoleic acid.

Based on the stimulatory effect of vaspin on NO bioavailability through the activation of eNOS in HAECs, we examined its effect in vascular tissue. As shown in Figure 2A, vaspin treatment increased the NO level by approximately 63% in conditioned media of vascular tissues as it did in HAECs. The pretreatment of L-NNA also abolished this effect of vaspin on NO in vascular tissues. In line with the stimulatory effects of vaspin on the phosphorylation of eNOS and VASP in HAECs (Figure 1B and 1C), vaspin treatment increased the serine sites’ phosphorylations of eNOS and VASP in vascular tissues as well. (Figure 2B and 2C).

In the background that vaspin increased the NO bioavailability through the activation of eNOS, we examined whether vaspin could reverse fatty acid induced endothelial dysfunction in aortic tissue using ex-vivo aortic ring study (Figure 2D). As reported previously [18], LA treatment significantly decreased endothelium-dependent vascular relaxation in the isolated aorta (Figure 2D). However, pretreatment of vaspin significantly inhibited LA-induced decrease in endothelium-dependent vasorelaxation compared to LA alone group (Figure 2D).

### Effects of Vaspin on the Level of ADMA and DDAH II Expression

**Figure 3:**
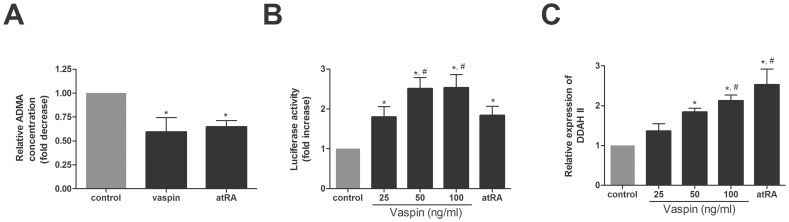
Vaspin decreases the level of ADMA in conditioned media of HAECs and increases DDAH II expression in HAECs. A. Effects of vaspin and atRA on ADMA levels in conditioned media of HAECs. HAECs were treated with vaspin (100 ng/ml) and atRA (1 µM) for 24 hr. **B–C.** The effect of vaspin on DDAH II promoter activity (B) and mRNA expression (C). DDAH II promoter activity and mRNA expression were measured at 16 hr after treatment with various concentrations of vaspin ranged from 25 to 100 ng/ml. atRA was used as a positive control. Data are shown as means ± SEM of at least three independent experiments. In A: ^*^ *p*<0.05 vs. untreated (Control). In B and C: ^*^ *p*<0.05 vs. untreated (Control); ^#^ *p*<0.05 vs. 25 ng/ml of vaspin group. ADMA, asymmetric dimethylarginine; atRA, all-trans-retinoic acid; DDAH II, dimethylarginine dimethylaminohydrolase II.

Compared to control cells, incubation of HAECs with 100 ng/ml of vaspin for 24 h decreased ADMA level by approximately 40% in conditioned media of HAECs (Figure 3A). This effect of vaspin on ADMA was also comparable to that of atRA (Figure 3A).

We next tested whether vaspin may decrease ADMA level by regulating the expression of DDAH II, an enzyme known to degrade ADMA. For this, we measured the changes in the mRNA expression and promoter activity of DDAH II after vaspin treatment. As shown in Figure 3, vaspin treatment increased DDAH II promoter activity as measured by luciferase assay (Figure 3B) and DDAH II mRNA expression as measured by real time PCR (Figure 3C).These results support the hypothesis that vaspin decreases ADMA levels by enhancing the production of DDAH II.

For further examination of the role of DDAH II in vaspin-induced changes in the levels of ADMA and NO, we examined the levels of them after the transfection with DDAH II siRNA. We could observe that vaspin regulates the levels of ADMA and NO as well as the eNOS activity in a DDAH II dependent manner (Figure S1).

### Vaspin Induces STAT3 Activation in Endothelial Cells

**Figure 4:**
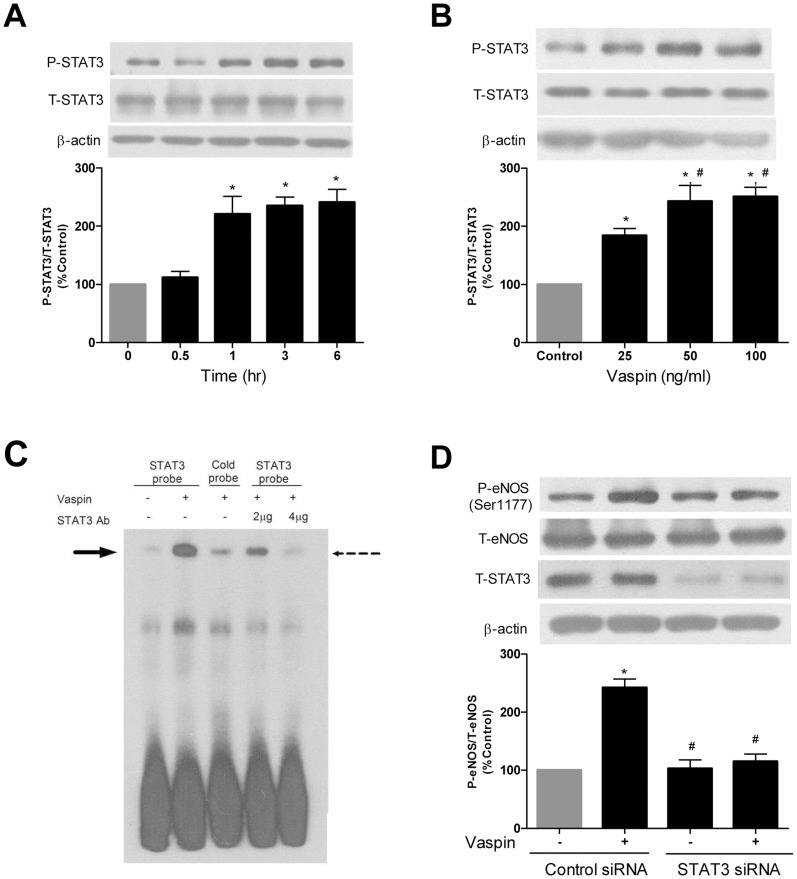
Vaspin increases eNOS activity in a STAT3-dependent manner. **A–B.** Effects of vaspin on STAT3 phosphorylation in HAECs according to the incubation time of vaspin (A) and dose of vaspin (B). HAECs were treated with 100 ng/ml of vaspin for the indicated time (A) and indicated concentrations of vaspin for 6 hr (B). **C** Effects of vaspin on STAT3-DNA binding activity. Electrophoretic mobility shift analysis (EMSA) was used to determine the DNA-binding activity of STAT3 in HAECs. Cells were treated with STAT3 probe or cold probe in the absence or the presence of 100 ng/ml of vaspin for 6 hr. Nuclear extracts from the treated and untreated control cells were isolated and used in an EMSA with biotin-labeled STAT3 oligonucleotide or cold probe. We used 2 ug and 4 ug of STAT3 Ab to identify the supershifts. The solid arrow and dashed arrow indicate the STAT3 binding complex and supershift, respectively. Data are representative results from three separate experiments. **D.** Effect of STAT3 siRNA on vaspin-induced eNOS phosphorylation in HAECs. HAECs were treated with control siRNA ± vaspin 100 ng/ml or STAT3 siRNA ± vaspin 100 ng/ml. Relative expression of STAT3 and eNOS were measured at 16 hr after vaspin treatment. Data are shown as means ± SEM of at least three independent experiments.In A: ^*^ *p*<0.05 vs. untreated (Control). In B: ^*^ *p*<0.05 vs. untreated (Control); ^#^ *p*<0.05 vs. 25 ng/ml of vaspin group. In D: ^*^ *p*<0.05 vs. control siRNA alone;^ #^ *p*<0.05 vs. control siRNA+vaspin. STAT3, signal transducer and activator of transcription 3; eNOS, endothelial nitric oxide synthase.

Next, we examined whether vaspin could activate STAT3, a nuclear transcription factor having binding sites at DDAH II promoter regions [11], to investigate whether STAT3 might contribute vaspin-induced reduction of ADMA and thereby increased eNOS activity. As shown in Figure 4A, incubation of HACEs with 100 ng/ml of vaspin induced a steady increase in STAT3 phosphorylation that peaked at 1 h and lasted until 6 h. Dose-response studies demonstrated that STAT3 phosphorylation was significantly increased after treatment with 25 ng/ml or more of vaspin compared to control cells (Figure 4B). Total STAT3 expression was unchanged after vaspin treatment. In addition, we performed EMSA to further demonstrate whether vaspin stimulated STAT3-DNA-binding activity. As shown in Figure 4C, vaspin increased DNA-binding activity of STAT3.

We further tested if vaspin may cause eNOS phosphorylation via STAT3 activation (Figure 4D). As shown in Figure 4D, the effect of vaspin on the increased phosphorylation of eNOS was abolished by the transfection of STAT3 siRNA. Collectively, these data suggest that vaspin increased eNOS activity in a STAT3-dependent manner. In line with this, we could observe that STAT3 mediated the vaspin-induced changes in the levels of NO and ADMA in conditioned media of HAECs. (Figure S2).

### STAT3 Mediates the Effect of Vaspin on DDAH II Promoter Activity

**Figure 5:**
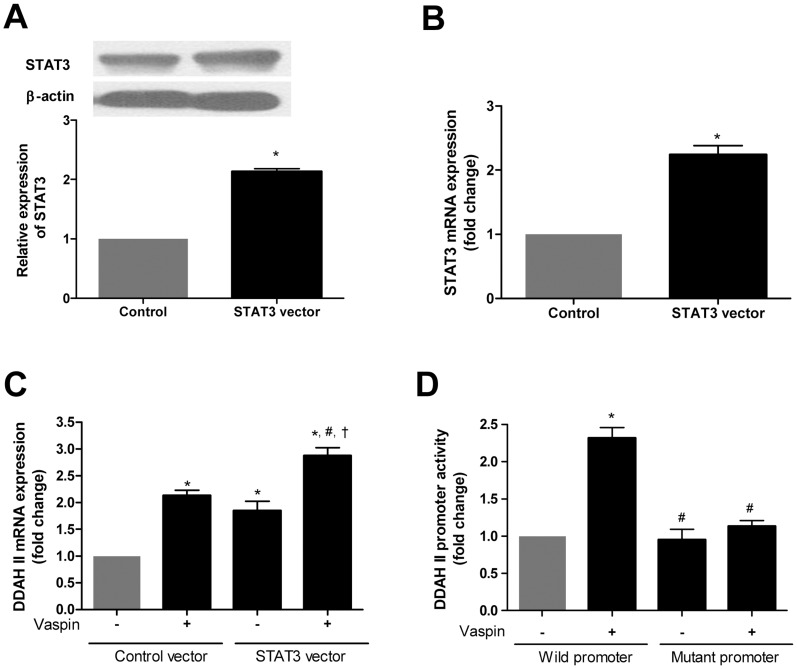
STAT3 mediates the effect of vaspin on DDAH II promoter activity. A–B. The effect of STAT3-expression vector on STAT3 expression. The expressions of STAT3 at the level of protein (A) and mRNA were measured after 24 hr of transfection (B). **C.** The stimulatory effects of vaspin on STAT3-induced DDAH II expression. HAECs were transfected with 200 ng of STAT3-expression vector or control empty vector and then incubated with or without vaspin (100 ng/ml) for 16 hr. **D.** The role of STAT3-binding site in vaspin’s stimulatory effect on DDAH II expression. HAECs were transfected with DDAH II promoter-reporter plasmid (wild type or mutant type at putative STAT3-binding site) and stimulated with vehicle or vaspin (100 ng/ml) for 16 hr. Data are shown as means ± SEM of at least three independent experiments. In A and B: ^*^ *p*<0.05 vs. control vector. In C: ^*^ *p*<0.05 vs. untreated (Control); ^#^ *p*<0.05 vs. vaspin+control vector group; ^†^ *p*<0.05 vs. STAT3 vector alone group. In D: ^*^ *p*<0.05 vs. wild promoter only group; ^#^ *p*<0.05 vs. wild promoter+vaspin group. STAT3, signal transducer and activator of transcription 3; DDAH II, dimethylarginine dimethylaminohydrolase II.

Given that vaspin induced STAT3 activation in HAECs and STAT3 is known to increase DDAH II expression [11], [19], we next examined whether activated STAT3 might mediate the stimulatory effect of vaspin on DDAH II expression. We firstly confirmed that transfection with STAT3-expressing vector increased both protein level and mRNA expression levels of STAT3 (Figure 5A and 5B). Like vaspin treatment, overexpression of STAT3 gene significantly increased the expression of DDAH II gene (Figure 5C). Vaspin treatment in STAT3-expressing cells further elevated transcriptional activity of DDAH II gene (Figure 5C). Consistently, the stimulatory effect of vaspin on DDAH II promoter activity was ameliorated in the cells expressing DDAH II promoter harboring the mutations in STAT3 binding sites (Figure 5D). When we tested the vaspin-induced changes in the expression of DDAH II mRNA after transfection of STAT3 siRNA in HAECs, vaspin’s stimulatory effect on DDAH II mRNA expression was abolished by the treatment of STAT3 siRNA (Figure S2). These data suggest that STAT3 is an important signaling molecule in vaspin mediated transcriptional regulation of DDAH II.

## Discussion

In the present study, we investigated the effects of vaspin on eNOS activity and NO bioavailability and examined whether STAT3/DDAH/ADMA system was involved in that action of vaspin. Our findings firstly demonstrate that vaspin is involved in the regulation of NO through its stimulatory effect on STAT3 and subsequent transcriptional regulation of DDAH II in vascular endothelial cells. Previous studies showed that vaspin possessed antiapoptotic effect in endothelial cells [10], anti-inflammatory effect [20], and anti-migratory effect in vascular smooth muscle cells [21]. Considering the results of both previous studies mentioned above and this one, we propose that vaspin plays a protective role in the pathogenesis of atherosclerosis.

There is an increasing amount of evidence supporting the pivotal role of ADMA, an endogenous NOS inhibitor, in a large number of disorders characterized by endothelial dysfunction [5], [22]–[24]. It is also apparent that DDAH is a critical modulator of intracellular and extracellular ADMA concentrations and has potential to regulate NO bioavailability through that modulation [7], [8], [22], [25]. In the present study, we investigated the effects of vaspin on eNOS activity and NO bioavailability and examined whether STAT3/DDAH/ADMA system was involved in that action of vaspin. We found that vapin has additional antiatherogenic effect on vascular endothelial cells besides its antiapoptotic effect against FFA [10].

We have previously shown that alpha-lipoic acid, a potent antioxidant used for diabetic neuropathy, enhanced DDAH activity and DDAH II gene expression through the regulation of STAT3 in cultured endothelial cells [26]. This is the background why we tried to examine vaspin’s action on eNOS activity and NO bioavailability through the STAT3/DDAH/ADMA system in cultured endothelial cells.

The main findings of this study were that (1) vaspin increased the levels of NO and eNOS activity; (2) vaspin decreased the levels of ADMA and activated DDAH II gene expression by increasing its promoter activity; (3) vaspin increased the phosphorylation of STAT3 and increased eNOS activity in a STAT3-dependent manner; (4) STAT3 mediated the effect of vaspin on DDAH II promoter activity. Overall, these results suggested that vaspin increased NO bioavailability through its stimulatory effect on STAT3 and thereby activating DDAH II gene expression with subsequent reduction of ADMA levels.

ADMA is an endogenous inhibitor of all types of nitric oxide synthases (NOSs) [4]. It has long been thought that the NOS inhibition of ADMA is attributable to its competitive inhibition as an L-arginine analogue [4]. However, it has been demonstrated that endothelial dysfunction caused by ADMA might be attributable to its inhibition of eNOS phosphorylation [27]. In this context, we measured the eNOS phosphorylation as a final phenomenon which is induced by vaspin’s stimulatory effect on STAT3/DDAH II/ADMA system.

Two isoforms of DDAH are known, DDAH I and DDAH II with distinct tissue distribution [23]. DDAH I is typically found in tissues expressing neuronal NOS, whereas DDAH II predominates in vascular tissues containing the eNOS. Since the main determinant of CVD is NO synthesis by eNOS [3], isoform-specific approach for DDAH II might be more favorable to understand the pathophysiology and to consider the therapeutic modulation.

The activity of DDAH appears to be controlled by complex regulatory mechanisms, which are not fully elucidated. There are some recent evidences that DDAH activity is impaired by oxidative stress, permitting ADMA to accumulate [7], [28]. Therefore, the strategies to reduce oxidative stress would likely enhance DDAH activity and thereby decrease ADMA levels. Indeed, several agents having anti-oxidant property such as taurine [29], vitamine E [29], pravastatin [30], and fenofibrate [31] were shown to increase DDAH activity. In previous studies in which the effect of vaspin on vascular smooth muscle cell was investigated [20], [21], and our study (Figure S3), vaspin reduced reactive oxygen species (ROS) generation. Although we did not measure the DDAH activity after treatment of vaspin, this anti-oxidant property of vaspin might contribute to the reduced level of ADMA and increased bioavailability of NO.

The nuclear transcription factor STAT3, a well known downstream signal of leptin [32], contributes to various physiologic processes via its regulatory function on gene expression [33]–[36]. It has been suggested that the binding site for STAT3 presents in the promoter region of DDAH II gene [11]. In this background, we investigated the role of STAT3 in increased DDAH II gene expression by vaspin. In this study, we showed that the mRNA expression of DDAH II was induced by STAT3 transfection in HAECs and vaspin amplified it (Figure 5C). Furthermore, the stimulatory effect of vaspin on the expression of DDAH II was not evident when the putative STAT3 binding site in DDAH II promoter region was mutated (Figure 5D), indicating a direct role of STAT3 in mediating the stimulatory signaling on DDAH II gene expression by vaspin. This hypothesis was supported by the data obtained from study on the binding affinity of STAT3 on DNA with or without vaspin (Figure 4C). However, the exact mechanism by which vaspin induces STAT3 activation is not clear by now. Additional studies are necessary to identify possible mechanisms underlying it.

STAT family genes are regulated by numerous of biological processes and have been implicated in a variety of cellular function [37]. There are seven known STATs (STAT 1, 2, 3, 4, 5a, 5b, and 6), of which STAT3 is mostly notably activated by the interleukin-6 (IL-6) family of cytokine and thereby exerting inflammatory responses [38]. The activated STAT3 dimerizes and translocates to the nucleus to activate or repress downstream target gene expression [37]. The zygotic STAT3 deletion is lethal before gastrulation [39]. Several genetic ablation studies using cell type-specific STAT3 knock out (KO) mice in keratinocyte [40], or macrophage/neutrophils [41] demonstrated that STAT3 played a critical role in controlling inflammation as the deletion of STAT3 causes an exacerbated inflammatory response. Regarding to vascular cells, several previous studies have demonstrated that endothelial STAT3 played an important protective role against oxidative injury [42], or ischemic injury [37], or endotoxin [43]. In the similar context, STAT3 might exert its protective effect in view of STAT3-mediated transcriptional regulation of DDAH II by vaspin.

The serum level of vaspin in human varies according to the characteristics of the studied subjects [44]–[47]. In view of atherosclerosis, the vaspin levels varied between studies [48], [49]. It has been suggested that elevated level of vaspin is a compensatory factor in the status of obesity or insulin resistance [9], [50], [51]. Considering the beneficial effect of vaspin on vascular cells demonstrated by previous studies [10], [20], [21], and this study, our result can be another evidence supporting that vaspin acts as a compensatory factor.

In conclusion, we identified vaspin, a recently identified adipocytokine, as a new transcriptional modulator of DDAH II and an activator of endothelial STAT3. These results provide a novel molecular mechanism underlying vaspin’s antiatherogenic function in endothelial cells. Further studies are needed to elucidate the specific mechanism by which vaspin activates STAT3 and its antiatherogenic function in vivo models.

## Supporting Information

Figure S1 **DDAH-II mediates the vaspin-induced changes in the levels of NO, ADMA and the eNOS activity in HAECs. A-B.** Effect of DDAH II siRNA on vaspin-induced changes in the levels of NO (A) and ADMA (B) in conditioned media of HAECs. HAECs were treated with control siRNA ± vaspin 100 ng/ml or DDAH II siRNA ± vaspin 100 ng/ml. Relative concentrations of NO and ADMA were measured at 24 hr after vaspin treatment. **C.** Effect of DDAH II siRNA on vaspin induced eNOS phosphorylation in HAECs. HAECs were treated with control siRNA ± vaspin 100 ng/ml or DDAH II siRNA ± vaspin 100 ng/ml. Relative expression of eNOS was measured at 16 hr after vaspin treatment. The blocked expression of DDAH II by DDAH II siRNA was confirmed by using the real time PCR for DDAH II mRNA in every experiment. Data are shown as means ± SEM of at least three independent experiments.^*^ *p*<0.05 vs. control siRNA alone;^ #^ *p*<0.05 vs. control siRNA+vaspin. DDAH II, dimethylarginine dimethylaminohydrolase II; NO, nitric oxide; ADMA, asymmetric dimethylarginine; eNOS, endothelial nitric oxide synthase.
(TIF)

Figure S2 **STAT3 mediates the vaspin-induced changes in the levels of NO, ADMA and the expression of DDAH II in HAECs. A-B.** Effect of STAT3 siRNA on vaspin-induced changes in the levels of NO (A) and ADMA (B) in conditioned media of HAECs. HAECs were treated with control siRNA ± vaspin 100 ng/ml or STAT3 siRNA ± vaspin 100 ng/ml. Relative concentrations of NO and ADMA were measured at 24 hr after vaspin treatment. **C.** Effect of STAT3 siRNA on vaspin induced DDAH II mRNA expression in HAECs. HAECs were treated with control siRNA ± vaspin 100 ng/ml or STAT3 siRNA ± vaspin 100 ng/ml. Relative expressions of STAT3 and DDAH II mRNA was measured at 16 hr after vaspin treatment. Data are shown as means ± SEM of at least three independent experiments.^*^ *p*<0.05 vs. control siRNA alone;^ #^ *p*<0.05 vs. control siRNA+vaspin. STAT3, signal transducer and activator of transcription 3; NO, nitric oxide; ADMA, asymmetric dimethylarginine; DDAH II, dimethylarginine dimethylaminohydrolase II.
(TIF)

Figure S3 **The antioxidant effect of vaspin against hydrogen peroxide (H_2_O_2_)-induced oxidative stress in HAECs.** Intracellular ROS generation was measured by a flow cytometry (FACSCaliber, Becton Dickinson, NJ, USA) using DCHF_2_-DA (Molecular Probes). **A.** The representative microphotographs of DCFH_2_-DA (green fluorescence) staining. The green fluorescence was visualized using a confocal microscopy (LSM710, ZEISS, Germany). Magnification, x 40. **B.** Quantification of fluorescence density was done using a flow cytometry. Data are shown as means ± SEM of at least three independent experiments. HAECs were treated with 100 ng/ml of vaspin 1 hr before the incubation of H_2_O_2_ for 15 min, which was followed by the incubation of 2.5 µmol/ml DCFH_2_-DA for 15 min. Fluorescence was measured at 30 min after the incubation of DCHF_2_-DA.
(TIF)

Methods S1 **Real time polymerase chain reaction (PCR) analysis.**
(DOCX)
