# Supplementary figures and images for: Vaspin Increases Nitric Oxide Bioavailability through the Reduction of Asymmetric Dimethylarginine in Vascular Endothelial Cells
Source: PLoS One. 2012 Dec 28;7(12):e52346. doi: 10.1371/journal.pone.0052346 (PMC3532208; doi:10.1371/journal.pone.0052346)

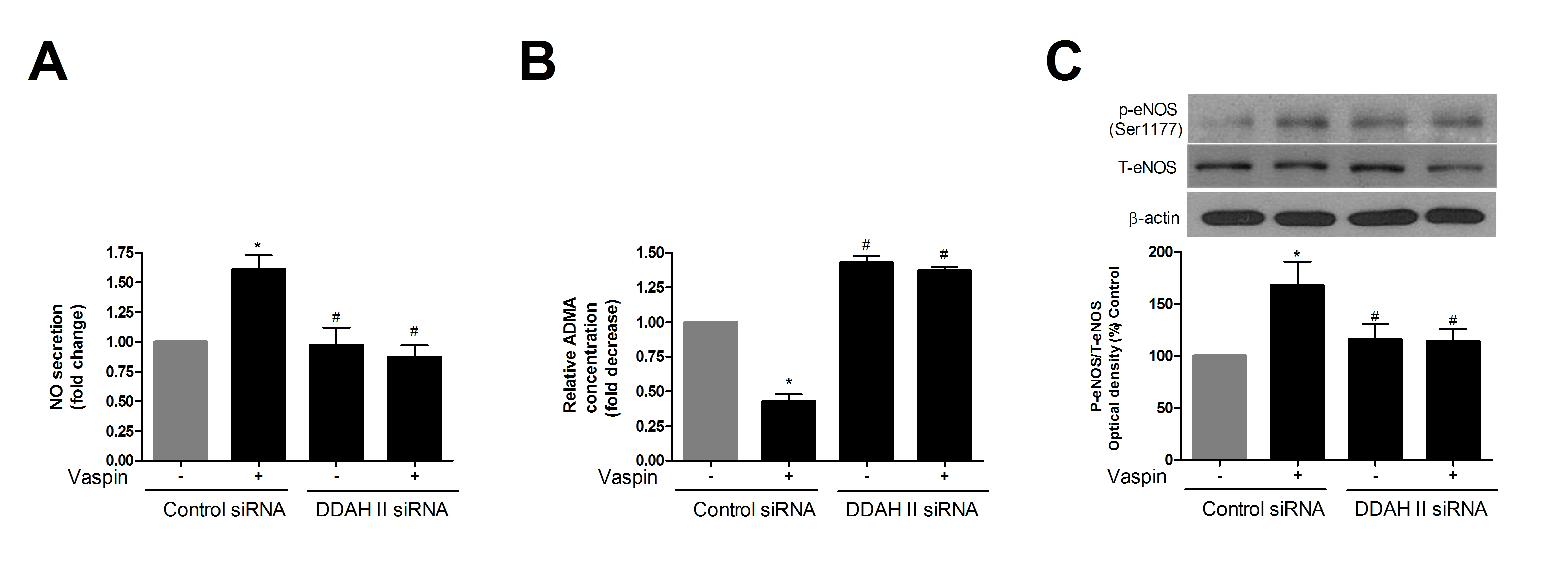

Supplement: Figure S1 — DDAH-II mediates the vaspin-induced changes in the levels of NO, ADMA and the eNOS activity in HAECs. A-B. Effect of DDAH II siRNA on vaspin-induced changes in the levels of NO (A) and ADMA (B) in conditioned media of HAECs. HAECs were treated with control siRNA ± vaspin 100 ng/ml or DDAH II siRNA ± vaspin 100 ng/ml. Relative concentrations of NO and ADMA were measured at 24 hr after vaspin treatment. C. Effect of DDAH II siRNA on vaspin induced eNOS phosphorylation in HAECs. HAECs were treated with control siRNA ± vaspin 100 ng/ml or DDAH II siRNA ± vaspin 100 ng/ml. Relative expression of eNOS was measured at 16 hr after vaspin treatment. The blocked expression of DDAH II by DDAH II siRNA was confirmed by using the real time PCR for DDAH II mRNA in every experiment. Data are shown as means ± SEM of at least three independent experiments.* p<0.05 vs. control siRNA alone; # p<0.05 vs. control siRNA+vaspin. DDAH II, dimethylarginine dimethylaminohydrolase II; NO, nitric oxide; ADMA, asymmetric dimethylarginine; eNOS, endothelial nitric oxide synthase. (TIF) [file pone.0052346.s001.tif]

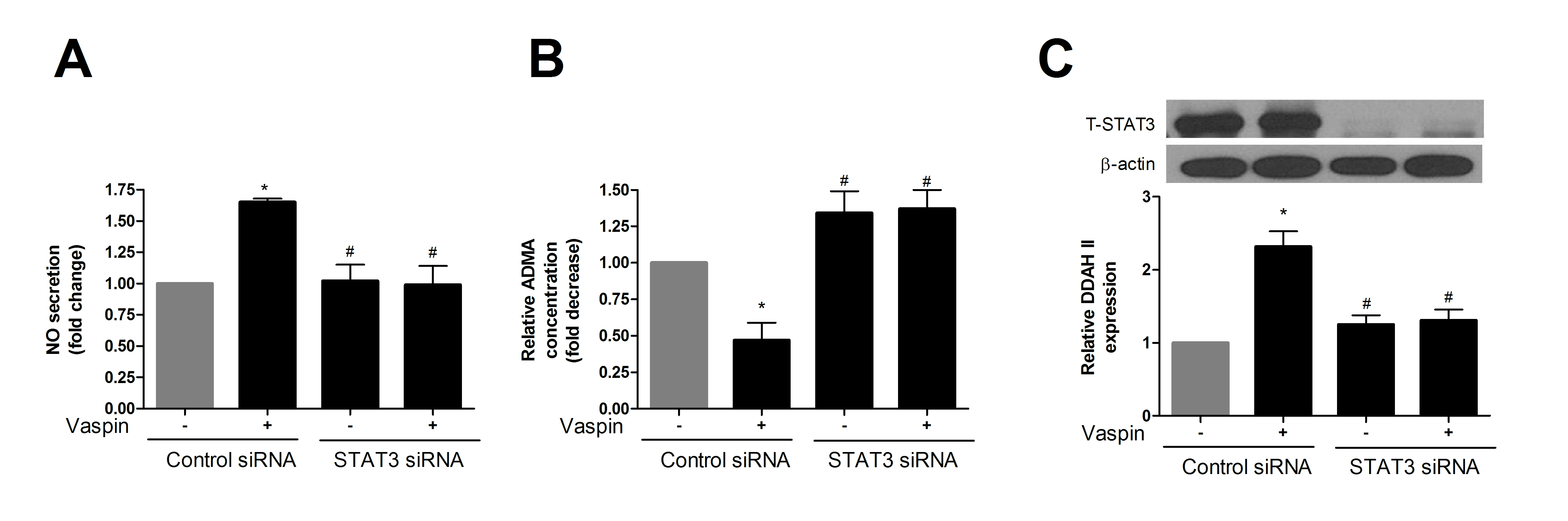

Supplement: Figure S2 — STAT3 mediates the vaspin-induced changes in the levels of NO, ADMA and the expression of DDAH II in HAECs. A-B. Effect of STAT3 siRNA on vaspin-induced changes in the levels of NO (A) and ADMA (B) in conditioned media of HAECs. HAECs were treated with control siRNA ± vaspin 100 ng/ml or STAT3 siRNA ± vaspin 100 ng/ml. Relative concentrations of NO and ADMA were measured at 24 hr after vaspin treatment. C. Effect of STAT3 siRNA on vaspin induced DDAH II mRNA expression in HAECs. HAECs were treated with control siRNA ± vaspin 100 ng/ml or STAT3 siRNA ± vaspin 100 ng/ml. Relative expressions of STAT3 and DDAH II mRNA was measured at 16 hr after vaspin treatment. Data are shown as means ± SEM of at least three independent experiments.* p<0.05 vs. control siRNA alone; # p<0.05 vs. control siRNA+vaspin. STAT3, signal transducer and activator of transcription 3; NO, nitric oxide; ADMA, asymmetric dimethylarginine; DDAH II, dimethylarginine dimethylaminohydrolase II. (TIF) [file pone.0052346.s002.tif]

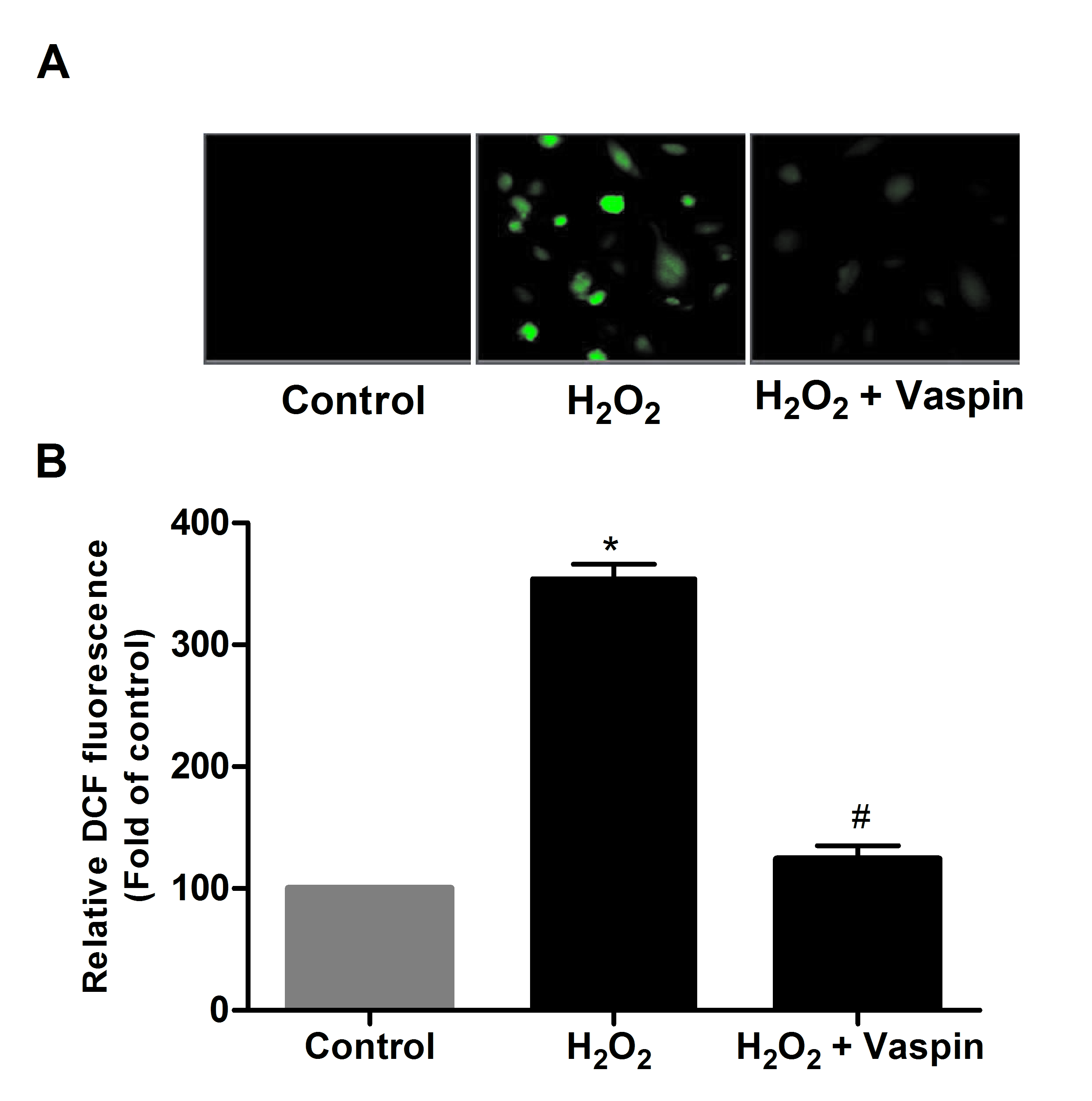

Supplement: Figure S3 — The antioxidant effect of vaspin against hydrogen peroxide (H2O2)-induced oxidative stress in HAECs. Intracellular ROS generation was measured by a flow cytometry (FACSCaliber, Becton Dickinson, NJ, USA) using DCHF2-DA (Molecular Probes). A. The representative microphotographs of DCFH2-DA (green fluorescence) staining. The green fluorescence was visualized using a confocal microscopy (LSM710, ZEISS, Germany). Magnification, x 40. B. Quantification of fluorescence density was done using a flow cytometry. Data are shown as means ± SEM of at least three independent experiments. HAECs were treated with 100 ng/ml of vaspin 1 hr before the incubation of H2O2 for 15 min, which was followed by the incubation of 2.5 µmol/ml DCFH2-DA for 15 min. Fluorescence was measured at 30 min after the incubation of DCHF2-DA. (TIF) [file pone.0052346.s003.tif]
