# Supplementary material for: Vaspin Increases Nitric Oxide Bioavailability through the Reduction of Asymmetric Dimethylarginine in Vascular Endothelial Cells
Source: PLoS One. 2012 Dec 28;7(12):e52346. doi: 10.1371/journal.pone.0052346 (PMC3532208; doi:10.1371/journal.pone.0052346)
Supplement: Methods S1 — Real time polymerase chain reaction (PCR) analysis. (DOCX) [file pone.0052346.s004.docx]

**Real time polymerase chain reaction (PCR) analysis**

cDNAs were synthesized using ReverTra ACE qPCR RT kit (Toyobo, Osaka, Japan). Real-time analysis was performed on an ABI 7500 Fast RT-PCR system (Foster City, CA, USA) with the SYBR Green kit (Applied Biosystems, CA, USA). Each sample was assayed in duplicate in a 20 μL reaction volume containing 1 μL cDNA (corresponding to 100 ng of total RNA input), 10 μL SYBR Green master mix (Applied Biosystems), and 1 μL of forward and reverse primer (10 pmole/μL for each). Negative controls (no template or RNA) were included to ensure the absence of contamination. Amplification of 18S rRNA was used as the internal control. Ratios of target gene and 18S rRNA expression levels were calculated using a relative quantification method (aka. ΔΔ Ct method) as described (User Bulletin No. 2, Applied Biosystems).

In brief, the amplification plot is the plot of fluorescence versus PCR number. The threshold cycle value (Ct) is the fractional PCR cycle number at which the fluorescent signal reached the detection threshold. Therefore, the input cDNA copy number and Ct are inversely related. Data were analyzed with the Sequence Detector System (SDS) software version 2.1 (ABI) and Ct value was automatically converted to fold change RQ value. The Fold change (RQ) = 2 ^− (ΔΔCT)^, where − (ΔΔCT) = − (ΔCT_trt_ − ΔCT_control_) = − [(Ct_Target_ – Ct18s)_trt_ − (Ct_Target_ – Ct18s)_control_].
